# Chief Complaint at Admission Relates to Troponin Level and Mortality in Patients With Non-ACS Troponin Elevation

**DOI:** 10.14740/jocmr2143w

**Published:** 2015-04-08

**Authors:** Eva Piscator, Lukas Lowing Svensson, Per Svensson

**Affiliations:** a Karolinska Institutet, Department of Medicine, Internal Medicine Unit and Emergency Department, Karolinska University Hospital Solna, Stockholm, Sweden; b Falun Hospital, Falun, Sweden

**Keywords:** Troponin, Non-ACS troponin elevation, Mortality

## Abstract

**Background:**

Elevated level of troponin T (TnT) in the absence of acute coronary syndrome (ACS) can be caused by a number of conditions but the relevance of the chief complaint at admission for TnT level and prognosis has not been reported previously. The aim was to study whether TnT level differs among chief complaints or underlying causes in patients with non-ACS TnT elevation and if these factors predict mortality.

**Methods:**

Patients admitted with TnT elevation were categorized as ACS or non-ACS and followed for 1 year. Statistical comparisons between different chief complaints and underlying causes were performed.

**Results:**

Patients with non-ACS TnT elevation (n = 71) were less likely to present with chest pain compared to ACS (n = 50) (37% vs. 74%, P < 0.001) whereas dyspnea (25%), syncope/arrhythmia (14%) or other chief complaints (24%) were more common. Patients with dyspnea and other chief complaints had higher peak values of TnT compared to chest pain (P < 0.05). The most common peak occurred within 3 hours after admission for chest pain, dyspnea and other chief complaints whereas for arrhythmia it occurred after 3 - 9 hours (P < 0.01). A peak value > 15 hours after admission was only observed among dyspnea and other chief complaints. Mortality was higher in patients presenting with dyspnea (50%) or other causes (35%) compared to chest pain (8%) or syncope/arrhythmia (10%) (P < 0.05). Renal failure was the only underlying cause that predicted mortality.

**Conclusion:**

Among patients with non-ACS TnT elevation, patients presenting with dyspnea had higher TnT and higher 1-year mortality, whereas patients with chest pain were at lower risk.

## Introduction

According to the universal definition of acute myocardial infarction (AMI), a typical increasing or decreasing pattern in troponin values has to be present to fulfill the diagnosis of AMI type 1 due to acute coronary syndrome (ACS) [1]. The sensitivity of troponin assays has increased and it has become more important to differentiate acute from chronic myocardial damage by evaluating the rise and fall of troponin concentration in serially drawn blood samples [1, 2]. Elevated troponin levels in the absence of ACS can be caused by a number of conditions [3-18]. In such conditions, the troponin levels are lower [5, 8, 9, 14-16, 18-20] with lower dynamics [2, 5, 18, 20] compared to troponin levels in ACS. To our knowledge, no study has compared troponin kinetics between different chief complaints at admission in non-ACS troponin elevation and few studies have compared the level [9, 16] and kinetics of troponin elevation among different underlying causes in non-ACS troponin elevation. The aim of this study was to investigate whether the level and kinetics of troponin T (TnT) in patients with non-ACS troponin elevation differed in relation to the chief complaint at admission and/or the adjudicated underlying cause of troponin elevation in an emergency ward population. A secondary aim was to study whether different chief complaints, troponin levels as well as the adjudicated underlying cause were predictors of 1-year mortality in this patient group.

## Methods

### Patients

The study group comprised all patients registered in RIKS-HIA, the Swedish national registry for acute coronary care, at the short-term emergency ward at Karolinska University Hospital Solna during the period from October 6, 2008 to January 31, 2010. Patients included in RIKS-HIA were all patients admitted from the emergency department to the ward where a repeated troponin analysis (one or consecutive) was planned to rule out ACS. In the event of more than one admission per patient during the study period, only the first admission was included. In total 488 patients were included.

Information in the registry was based on the patient’s medical record from which information including age, sex, weight, height, smoking status, former and present diseases, diagnosis, results from laboratory sampling, diagnostic examinations, chief complaint and time for admission had been collected. The first result from the laboratory tests during the hospital stay was recorded in RIKS-HIA. During the study period, information to RIKS-HIA was reported by the doctor in charge of the patient and nurses responsible for the registry in the emergency ward, according to a specific RIKS-HIA case report form [21]. In the event of incomplete reporting, data were supplemented from the medical record. Data extracted from RIKS-HIA contained information on the chief complaint at admission in three categories (chest pain, dyspnea or other). In all patients with a chief complaint in the category “other”, the most significant symptom that caused the patient to seek treatment was extracted from the admission notes in the medical journal. A new category “syncope/arrhythmia” was created for all the different chief complaints thought to relate to a circulatory cause comprising the following: syncope, presyncope, collapse, arrhythmia and palpitation. Information regarding all consecutive sampling of TnT during the hospital stay was gathered from the medical record and the results, date and time (HH.MM) were registered. The time in hours and minutes between the time of sampling and the time of admission was calculated for each result. Troponin data were categorized into the following four categories: 0 - 3 h, 3 - 9 h, 9 - 15 h and > 15 h after admission. Peak TnT was calculated as the highest recorded value independent of the number of samples taken; if the highest value was the same in consecutive sampling, the first was used as the maximum.

### Laboratory measurements

TnT was assayed with Elecsys TnT STAT (Roche Diagnostics GmbH, Mannheim, Germany) with a lower detection limit of 0.01 μg/L. The cut off for myocardial infarction was 0.03 μg/L (99th percentile value). Values < 0.01 μg/L from the laboratory were listed in the analysis as 0 μg/L unless the TnT value was used in the calculation of TnT kinetics in which case the value was listed as 0.005 μg/L.

### Classification of the TnT elevation

To classify the cause of the TnT elevation, all medical records were reviewed and patients were categorized into five groups: no TnT elevation (TnT < 0.01 μg/L); non-significant TnT elevation (TnT 0.01 - 0.02 μg/L); ACS: TnT elevation (TnT > 0.03 μg/L) with ACS; non-ACS TnT elevation: TnT elevation (TnT > 0.03 μg/L) without ACS and TnT elevation (TnT > 0.03 μg/L) of indeterminate origin. TnT elevation with ACS was classified in accordance with the universal definition of myocardial infarction type 1 [1]: TnT > 0.03 μg/L in conjunction with symptoms of ischemia; ECG changes indicative of new ischemia; development of pathological Q waves in the ECG; imaging evidence of new loss of viable myocardium or new regional wall motion abnormality. TnT > 0.03 μg/L with the absence of these features, together with a clinical picture of alternative cause was classified as non-ACS TnT elevation. In cases where more than one cause of TnT elevation was possible, all were stated. If the review could not state that the TnT elevation was caused either by ACS or not by ACS or if no probable cause could be stated, the cause of the TnT elevation was categorized as indeterminate. The review was carried out by a medical student or a resident medical officer in internal medicine with a second review by a specialist in internal medicine and cardiology if the primary reviewer disagreed with the cause/causes of the TnT elevation stated in the medical records, or if the cause of the TnT elevation was not clearly expressed. Discrepancies were settled by mutual consensus. In order to facilitate the analysis, the underlying cause was categorized into the following five subgroups: renal; respiratory comprising pneumonia, exacerbation of chronic obstructive pulmonary disease, asthma, respiratory insufficiency, hypoxia; infection comprising pneumonia, infection, sepsis, bronchitis; arrhythmia comprising atrial fibrillation/flutter, supraventricular tachycardia, bradyarrhythmia or unspecified arrhythmia and structural heart disease comprising congestive heart failure, vitium organicum cordis and left ventricular hypertrophy.

### Survival data

The primary outcome variable for the follow-up analysis was time from admission to the date of mortality. Data on all-cause mortality were obtained from the Swedish national population registry. All patients were followed until the date of mortality or to the end of follow-up after 1 year (365 days).

### Statistical analysis

The results are presented as mean with standard deviations or median with 25 - 75 percentiles. Student’s *t*-tests or the Mann-Whitney U-test were used for independent samples when appropriate. The maximum likelihood Chi-square test was used to compare the distribution of categorical variables between groups. As the distribution of TnT was skewed, the Kruskal-Wallis one-way ANOVA was used to compare the groups with different chief complaints at admission. The cumulative proportion surviving in the different categories of TnT elevation and the chief complaints were illustrated using Kaplan-Meier curves and the log-rank test was used to compare mortality between the different groups. In non-ACS TnT elevation, the predictive value of clinically relevant variables from patient history and demography, category of chief complaint, category of adjudicated cause for TnT elevation, TnT level, eGFR as well as logCRP on all-cause mortality at 1 year was assessed by univariate Cox regression analysis. As a comparison, a similar analysis was performed in the group with ACS. A P-value less than 0.05 was considered significant. Statistical analysis and database management were performed using StatSoft, Inc. (2013), Statistica (data analysis software system), version 12 (www.statsoft.com).

### Ethical approval

The study was approved by the Regional Ethical Review Board in Stockholm.

## Results

Out of the 488 patients included, 325 (67%) had no TnT elevation and 33 (7%) had a non-significant TnT elevation. Out of the 130 troponin-positive patients, 50 (38%) were classified as ACS, 71 (55%) as non-ACS TnT elevation, eight (2%) as TnT elevation of indeterminate origin and one patient had a procedure-related myocardial infarction. Baseline characteristics are presented in Table 1. When compared to patients with ACS, patients with non-ACS TnT elevation more frequently had congestive heart failure and/or atrial fibrillation/flutter, lower blood pressure and eGFR, were hospitalized fewer days and to a lesser extent transferred to the coronary care unit for further medical care. The different chief complaints at admission among the groups are presented in Table 2. Chest pain was the most common presenting symptom in all categories but the distribution of chief complaints differed between patients with non-ACS TnT elevation and those with ACS (P < 0.001).

**Table 1:** Baseline Characteristics

	ACS (n = 50)	Non-ACS TnT-el (n = 71)	Non-sign TnT-el (n = 33)	TnT-el of indet orig (n = 8)	No TnT-el (n = 325)
Age (years)	74 ± 14	77 ± 12	70 ± 15	76 ± 13	64 ± 14
Female	21 (42)	28 (39)	12 (36)	6 (75)	165 (51)
Current smoker	8 (16)	10 (14)	4 (12)	1 (13)	58 (18)
Previous	13 (26)	25 (35)	17 (52)	3 (38)	94 (29)
CHF	14 (28)*	36 (51)	8 (24)	0	25 (8)
CAD	25 (50)	35 (49)	15 (45)	2 (25)	90 (28)
Hypertension	24 (48)	44 (62)	21 (64)	4 (50)	157 (48)
Diabetes	13 (26)	25 (35)	7 (21)	3 (38)	39 (12)
BMI (kg/m^2^)	26.3 ± 5.6	24.9 ± 4.7	25.8 ± 4.9	25 ± 2.9	27.1 ± 5.0
AF on arrival	6 (12)*	23 (32)	10 (30)	1 (13)	34 (10)
HF (beats/min)	91 ± 26	96 ± 30	82 ± 30	88 ± 28	78 ± 19
SBP (mm Hg)	152 ± 30	141 ± 33	148 ± 33	155 ± 18	154 ± 29
DBP (mm Hg)	86 ± 19*	77 ±18	80 ± 16	86 ± 13	83 ± 15
eGFR (mL/min/1.73^2^)	61 ± 30*	49 ± 26	56 ± 24	57 ± 24	77 ± 20
Hb (g/L)	131±29	126 ± 21	132 ± 16	136 ± 20	139 ± 15
CRP (mg/L)	6 (2 - 36)	14 (4 - 42)	7 (3 - 19)	6 (2 - 8)	2 (0 - 4)
Glucose (mmol/L)	7.5 (6.1 - 11.2)	7 (5.7 - 9.2)	6.9 (6.1 - 8.1)	9.4 (6.9 - 15.1)	6.1 (5.4 - 7.1)
Transferral CCU	29 (58)**	16 (23)	5 (15)	5 (63)	27 (8)
Hospitalization (days)	4 (3 - 7)**	2.5 (1 - 4.5)	2 (1 - 4)	4 (2 - 10)	1 (0.5 - 1)

Values are mean ± SD, median (IQR) or number (%). El: elevation; sign: significant; indet: indeterminate; orig: origin; CHF: congestive heart failure; CAD: former percutaneous coronary intervention and/or coronary artery bypass grafting and/or acute myocardial infarction; BMI: body mass index; AF: atrial fibrillation/flutter; PM: pacemaker; HF: heart frequency; SBP: systolic blood pressure; DBT: diastolic blood pressure; eGFR: estimated glomerular filtration rate according to MDRD; Hb: hemoglobin; CRP: C-reactive protein; CCU: coronary care unit. *P < 0.05 vs. non-ACS TnT-el. **P < 0.001 vs. non-ACS TnT-el.

**Table 2:** Chief Complaints at Admission

Chief complaints	ACS (n = 50)	Non-ACS TnT-el (n = 71)	Non-sign TnT-el (n = 33)	TnT-el of indet orig (n = 8)	No TnT-el (n = 325)
Chest pain	37 (74)*	26 (37)	24 (73)	6 (75)	268 (82)
Dyspnoea	8 (16)*	18 (25)	4 (12)	1 (13)	9 (3)
Syncope/arrhythmia	3 (6)*	10 (14)	4 (12)	0	42 (13)
Others	2 (4)*	17 (24)	1 (3)	1 (13)	6 (2)

Values are number (%). One patient with a procedure related acute myocardial infarction is not presented in the table. El: elevation; sign: significant; indet: indeterminate; orig: origin. *P < 0.001 vs. non-ACS TnT-el.

**Figure 1:**
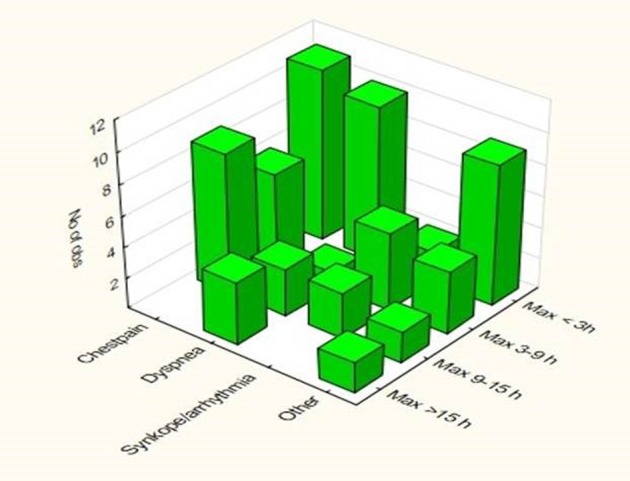
Distribution of the peak TnT level among four time categories after admission in relation to the different chief complaints.

In the 71 patients with non-ACS TnT elevation, a total of 218 TnT-samples had been analyzed (one in two patients, two in 15 patients, three in 34 patients, four in 16 patients, five in four patients). TnT kinetics for the 71 patients with non-ACS TnT elevation and for the different chief complaints at admission are presented in Table 3. Patients with dyspnea and other chief complaints at admission had higher peak values of TnT whereas peak values were lower in patients with chest pain (P < 0.05). The distribution in time for the TnT-peak value differed among the different chief complaints (P < 0.05) (Fig. 1). In patients with chest pain, dyspnea and other chief complaints, the most common peak occurred less than 3 h after admission whereas for arrhythmia the most common peak was observed 3 - 9 h after admission. A maximum value over 15 h after admission was observed only among patients admitted with dyspnea and other chief complaints.

**Table 3:** TnT Kinetics in Patients With ACS, Non-ACS TnT Elevation and Different Chief Complaints in Patients With Non-ACS TnT Elevation

Troponin categories	ACS (n = 50)	Non-ACS TnT-el (n = 71)	P-value	Non-ACS TnT-el (n = 71)				
				Chest pain (n = 26)	Dyspnea (n = 18)	Syncope/arrhythmia (n = 10)	Others (n = 17)	P-value
TnT < 3 h (μg/L)	0.05 (0.01 - 0.14) (44)	0.05 (0.03 - 0.08) (66)		0.04 (0.03 - 0.06) (25)	0.06 (0.04 - 0.08) (17)	0.04 (0.02 - 0.05) (9)	0.06 (0.03 - 0.15) (15)	0.18
TnT 3 - 9 h (μg/L)	0.23 (0.10 - 0.51) (37)	0.05 (0.04 - 0.11) (60)		0.04 (0.02 - 0.08) (23)	0.07 (0.05 - 0.1) (13)	0.05 (0.04 - 0.14) (10)	0.07 (0.04 - 0.14) (14)	0.19
TnT 9 - 15 h (μg/L)	0.26 (0.1 - 0.97) (22)	0.06 (0.04 - 0.13) (50)		0.04 (0.03 - 0.1) (21)	0.08 (0.06 - 0.13) (15)	0.06 (0.04 - 0.13) (9)	0.07 (0.06 - 0.15) (5)	0.16
TnT > 15 h (μg/L)	0.55 (0.20 - 1.32) (24)	0.10 (0.04 - 0.3) (15)		0.03 (0.03 - 0.03) (2)	0.10 (0.05 - 0.37) (7)	0.03 (0.03 - 0.03) (1)	0.23 (0.10 - 0.3) (5)	0.07
Max TnT (μg/L)	0.31 (0.12 - 0.83) (50)	0.06 (0.04 - 0.12) (71)	< 0.001	0.04 (0.03 - 0.07) (26)	0.08 (0.06 - 0.12) (18)	0.06 (0.04 - 0.14) (10)	0.08 (0.04 - 0.16) (17)	0.04
Time to max TnT (h:min)	8:19 (3:01 - 18:21)	6:02 (0:31 - 11:02)	< 0.001	6:10 (0:55 - 10:17)	2:29 (0:24 - 13:14)	6:57 (3:56 - 11:02)	0:57 (0:19 - 8:59)	0.72
No. of max < 3 h	12 (24)	32 (45)		11 (42)	10 (56)	2 (20)	9 (53)	
No. of max 3 - 9 h	13 (26)	16 (23)		6 (23)	1 (6)	5 (50)	4 (24)	
No. of max 9 - 15 h	7 (14)	17 (24)		9 (34)	3 (17)	3 (30)	2 (12)	
No. of max > 15 h	18 (36)	6 (9)	< 0.001	0	4 (22)	0	2 (12)	0.02

Values are median (IQR) (number of measurements) or number (%). P-value denotes comparison between ACS and non-ACS TnT-el with Mann-Whitney U-test or between all groups with Kruskall-Wallis ANOVA or maximum likelihood Chi-square.

Out of the 71 patients with non-ACS TnT elevation, 26 had one underlying cause, 34 had two underlying causes and 11 had more than two underlying causes. The most common underlying cause was renal failure (n = 43), followed by structural heart disease (n = 27), arrhythmia (n = 16) and respiratory disease (n = 13). The general characteristics and TnT kinetics for the different causes are shown in Supplementary 1 (www.jocmr.org). Infection was the only cause with significantly higher troponin levels and a longer time from admission to maximum. A later peak was more common in patients with infection and arrhythmia compared to patients without these causes. Patients with renal failure had significantly lower decrease from maximum TnT to the next value but did not differ in other aspects.

**Figure 2:**
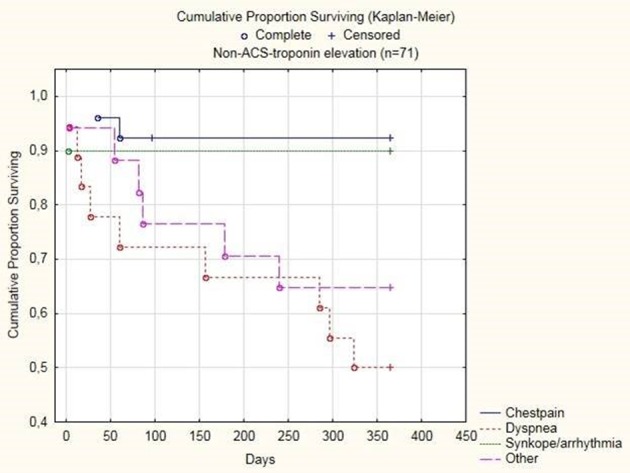
Cumulative proportion surviving among non-ACS troponin elevation in relation to chief complaint at admission.

One-year mortality was higher in all groups with troponin elevation compared to those with no elevation (P < 0.001) but did not differ between patients with ACS (32%) compared to non-ACS TnT elevation (27%). Among patients admitted with chest pain as the chief complaint, mortality was higher in ACS patients (34%) compared to in non-ACS TnT elevation (8%) (P < 0.05). Hazard ratios for the possible predictors of all-cause mortality studied are presented in Table 4. In non-ACS TnT elevation with chest pain and dyspnea as the chief complaint, the adjudicated cause being renal failure, eGFR as well as logCRP predicted 12-month mortality whereas troponin level or kinetics did not. Conversely, among patients with ACS: age, previous AMI and congestive heart failure as well as troponin level predicted mortality whereas the chief complaint at admission did not. The cumulative proportion surviving in patients with non-ACS TnT elevation in relation to different chief complaints is illustrated in Figure 2. One-year mortality was higher in patients presenting with dyspnea (50%) or other causes (35%) compared to those presenting with chest pain (8%) (P < 0.01 and P < 0.05 respectively). Presence of renal failure was the only cause that significantly predicted mortality.

**Table 4:** Univariate Predictors of 1-Year Mortality in Patients With ACS and Non-ACS TnT Elevation

	ACS (n = 50)		Non-ACS-TnT elevation (n = 71)	
	Hazard ratio (95% CI)	P-value	Hazard ratio (95% CI)	P-value
Age (years)	2.68 (1.34 - 5.36)	0.005	1.66 (0.86 - 3.22)	0.13
Male gender	0.19 (0.04 - 0.90)	0.03	3.5 (0.77 - 16.0)	0.10
Diabetes	1.00(0.21 - 4.82)	0.99	0.67 (0.18 - 2.50)	0.56
Hypertension	0.36 (0.09 - 1.43)	0.15	0.32 (0.10 - 1.07)	0.06
Previous AMI	11.1 (1.4 - 89.3)	0.02	1.03 (0.33 - 3.21)	0.95
CHF	8.4 (2.08 - 34.2)	0.003	2.12 (0.64 - 7.05)	0.22
Current smoking	*	0.99	1.94 (0.53 - 7.19)	0.32
Ever smoking	0.25 (0.05 - 1.19)	0.08	0.66 (0.21 - 2.05)	0.47
eGFR	0.49 (0.29 - 0.82)	0.007	0.55 (0.31 - 0.96)	0.03
logCRP	2.5 (1.6 - 4.0)	< 0.001	2.2 (1.3 - 3.8)	0.004
Max TnT	1.29 (1.08 - 1.54)	0.005	0.51 (0.03 - 9.5)	0.66
Abs increase	1.24 (0.92 - 1.67)	0.15	0.08 (0.00 - 87)	0.49
Abs decrease	0.81 (0.64 - 1.04)	0.09	0.60 (0.08 - 4.6)	0.62
Chief complaint				
Chest pain	2.7 (0.61 - 12.0)	0.18	0.19 (0.04 - 0.82)	0.03
Dyspnea	*	0.99	3.5 (1.4 - 8.7)	0.01
Arrhythmia	0.97(0.13 - 7.3)	0.97	0.3 (0.05 - 2.6)	0.29
Other	2.6 (0.3 - 19.7)	0.36	1.6 (0.6 - 4.4)	0.31
Cause				
Renal failure			3.8 (1.1 - 13.0)	0.04
Structural heart disease			1.4 (0.6 - 3.6)	0.44
Arrhythmia			0.7 (0.2 - 2.5)	0.60
Respiratory disease			1.2 (0.4 - 3.6)	0.75
Infection			2.1 (0.8 - 6.0)	0.15

Hazard ratios (HR) apply for an SD increase in continuous variables and for yes compared to no for all categorical variables. *Non-significant, HR and CI.

## Discussion

In this study, we investigated the relationship between the chief complaint at admission and the level of TnT and mortality in patients admitted with non-ACS TnT elevation. Maximum TnT was higher in patients with dyspnea or other chief complaints at admission compared to those with chest pain and arrhythmia. Patients with non-ACS TnT elevation presenting with dyspnea constituted a high-risk group with respect to 1-year mortality, as did patients with underlying renal failure, whereas patients with chest pain were at a lower risk.

To our knowledge, no previous study has reported on the kinetics of non-ACS related troponin elevation after admission for different chief complaints. One explanation of the higher troponin levels in groups admitted with dyspnea and other chief complaints compared to chest pain may be that chest pain is considered a more typical presentation of ACS. It is probable that the threshold of troponin elevation that is used to classify a patient as ACS is lower in chest pain compared to other chief complaints that are considered more atypical presentations of ACS.

The 1-year mortality in patients with non-ACS TnT elevation and dyspnea as the chief complaint in the current study was 50%, which implies that these patients constitute a group at very high risk. A previous study of patients that presented to the emergency department with dyspnea reported a 1-year mortality rate of 39% in patients with elevated TnT whereas it was 9% in patients with undetectable TnT [22]. In the present study, dyspnea at admission was a strong predictor for mortality, which is a novel finding within a non-ACS TnT elevation population. Conversely, among patients with non-ACS TnT elevation, 1-year mortality was lower in those admitted with chest pain. Although our findings need confirmation in further studies, this may have a significant clinical relevance. Patients with non-ACS TnT elevation constitute a very heterogeneous group and within this population there is a need for better risk stratification. Although information on the chief complaint at admission is usually easily available, knowledge of how to relate this information to future events is limited. Our findings support the use of chief complaint at admission as a risk-prediction tool among these patients.

In our study, the 1-year survival rate was similar in ACS and non-ACS TnT elevation and this finding differs from several previous studies in which mortality was higher in non-ACS troponin elevation [5, 8, 9, 15, 16]. In two of the studies, all patients in the hospital in which elevated troponin was recorded were included, thus also including postoperative and critically ill patients [8, 16]. Javed et al included all patients with more than one positive troponin finding routinely drawn in the hospital for all patients with certain chief complaints [15]. Ilva et al included patients with a presumptive diagnosis of ACS and troponin elevation at admission to the ED [5] and Wong et al included all patients admitted to the hospital with a presumptive diagnosis of ACS and one or more troponin blood tests [9]. In the current study only patients admitted to the short-term ward with a primary suspicion of ACS were included and patients admitted directly to the coronary care unit were not included. The ACS-patients included in this study probably represent a subgroup of ACS-patients that are elderly and with more co-morbidities. In this study, the level of troponin predicted 1-year mortality in the ACS study group but not in the non-ACS troponin elevation group. Our finding confirms previous studies in ACS that have shown a linear correlation between troponin level and mortality [2, 23, 24]. Our finding in the non-ACS troponin elevation group is in contrast to a previous study in which higher troponin level also among patients without ACS was an independent predictor of mortality [9]. In that study, a troponin elevation was defined as above or equal to 0.01 μg/L whereas in our study, for patients with a troponin level between 0.01 and 0.02 μg/L it was defined as non-significant troponin elevation. Since this group had a better prognosis compared to the non-ACS troponin elevation group, the different classifications may explain the different findings of the two studies.

The cause of troponin elevation was adjudicated based on a structured review of the clinical evidence in the medical files with a similar methodology as in previous studies. In the current study, the most common causes were renal failure followed by structural heart disease and arrhythmia. In two previous studies, causes of troponin elevation have been investigated in patients with normal coronary angiograms [6, 25]. In both of these studies, the most common cause was tachyarrhythmia, which occurred in 25-30% of the studied population, which is similar to a proportion of 23% with arrhythmia in the current study. A major difference is that troponin I was measured in the previous two studies whereas TnT was measured in the current one. TnT is more frequently elevated in renal failure compared to troponin I [26] which might explain the difference. Wong et al reported the primary diagnosis in 118 hospitalized patients without ACS with raised TnT [9]. The most common diagnoses were respiratory disease followed by CHF and supraventricular arrhythmia all of which were also common causes in the current study.

eGFR predicted mortality at 1 year in both ACS and non-ACS troponin elevation and, accordingly underlying renal failure as a cause for non-ACS troponin elevation was also a predictor for mortality. The relationship between chronic kidney disease and adverse outcomes including mortality is well established in ACS [27] and a similar relationship has been reported in a population of both ACS and non-ACS TnT elevation [16]. Further, elevated TnT is a predictor for mortality in stable patients with end-stage renal disease without cardiac symptoms [28]. CRP was a predictor of mortality in both ACS and non-ACS which confirms previous studies both in ACS [29, 30] and in chest pain-patients [30].

The study has some limitations. Only patients admitted primarily to the short-term emergency ward with suspected ACS were included and some selection bias is present. All patients with ST-elevation myocardial infarction and many patients with non ST-elevation myocardial infarction were admitted directly to the coronary care unit and were not included in this study. Also, patients with non-ACS troponin elevation may have been directly admitted to the coronary care unit and thus not included. The distinction between ACS and troponin elevation relating to other conditions is difficult. Diagnosis was performed retrospectively and there is a risk of misclassification between troponin elevation due to ACS and to non-ACS on the grounds of lack of available information. Further, there is also a risk of possible bias. Moreover, relatively few coronary angiograms were performed. Efforts were made to resolve all unclear cases by consensus. If a possible underlying cause was identified within the non-ACS-group, all such causes were stated and no judgment was made retrospectively regarding how much a specific cause contributed in a specific case. The study population was relatively small; however, it was well characterized regarding many variables. Further, we included all consecutively admitted patients to the short-term ward with suspicion of ACS during the study period. Thus a representative study group with non-ACS troponin elevation was included as well as relevant control groups with and without disease.

### Conclusion

Among patients admitted with non-ACS TnT elevation, maximum TnT was higher in patients with dyspnea or other chief complaints compared to those with chest pain and chief complaints related to arrhythmia. Patients with non-ACS TnT elevation presenting with dyspnea constituted a high-risk group with respect to 1-year mortality, as did patients with underlying renal failure, whereas patients with chest pain were at lower risk. Among patients with non-ACS-troponin elevation, chief complaint at admission may be used as a risk prediction tool; however, further studies are needed.

## Abbreviations

ACS: acute coronary syndrome
TnT: troponin T
eGFR: estimated glomerular filtration rate
